# Trehalose 6-phosphate signalling and impact on crop yield

**DOI:** 10.1042/BST20200286

**Published:** 2020-10-02

**Authors:** Matthew J. Paul, Amy Watson, Cara A. Griffiths

**Affiliations:** Plant Science, Rothamsted Research, Harpenden, Hertfordshire AL5 2JQ, U.K.

**Keywords:** crops, plant signal transduction, trehalose 6-phosphate

## Abstract

The domestication and breeding of crops has been a major achievement for mankind enabling the development of stable societies and civilisation. Crops have become more productive per unit area of cultivated land over the course of domestication supporting a current global population of 7.8 billion. Food security crops such as wheat and maize have seen large changes compared with early progenitors. Amongst processes that have been altered in these crops, is the allocation of carbon resources to support larger grain yield (grain number and size). In wheat, reduction in stem height has enabled diversion of resources from stems to ears. This has freed up carbon to support greater grain yield. Green revolution genes responsible for reductions in stem height are known, but a unifying mechanism for the active regulation of carbon resource allocation towards and within sinks has however been lacking. The trehalose 6-phosphate (T6P) signalling system has emerged as a mechanism of resource allocation and has been implicated in several crop traits including assimilate partitioning and improvement of yield in different environments. Understanding the mode of action of T6P through the SnRK1 protein kinase regulatory system is providing a basis for a unifying mechanism controlling whole-plant resource allocation and source-sink interactions in crops. Latest results show it is likely that the T6P/SnRK1 pathway can be harnessed for further improvements such as grain number and grain filling traits and abiotic stress resilience through targeted gene editing, breeding and chemical approaches.

## The discovery of T6P signalling in plants

A series of papers in the late 1990s and early 2000s began to indicate a widespread role for trehalose metabolism in plants [1–4]. Up until this time trehalose had been associated with more specialised drought-resilient resurrection plants and was not thought a central part of physiological processes [5]. Trehalose and trehalose 6-phosphate (T6P) were below the detection limit of systems used to measure plant carbohydrates and metabolites at that time. The prevailing view was that sucrose rather than trehalose was the dominant non reducing disaccharide in plants formed in photosynthesis as the major carbon source for growth, development and biosynthesis of end-products — starch, cell walls, protein and oil. How the use of sucrose was coordinated with growth and development was, however, unknown.

Heterologous expression of trehalose-synthesising genes in transgenic plants proved a turning point [6]. This was motivated by the desire to engineer drought-resilient crops and explore the possibility of using plants as a vehicle for trehalose production. Trehalose is synthesised in a two-step pathway in plants from UDP-glucose and glucose 6-phosphate, forming T6P first via trehalose phosphate synthase (TPS) and then converting T6P to trehalose via trehalose phosphate phosphatase (TPP). The phenotypes of the transgenic tobacco expressing the *E. coli* trehalose phosphate synthase (*otsA)* or trehalose phosphate phosphatase (*otsB)* genes were very striking showing robust shoots with changes in leaf area and photosynthetic capacity and improvements in productivity [7], atypical of transgenic plants modified in carbon metabolism, which are often severely impaired, or show no phenotype — at least in transgenics produced around this time. This gave an indication that some component of the trehalose pathway could be engaging with an endogenous system that regulated growth and development and, possibly given trehalose is a sugar, integrated sugar metabolism with growth and development.

**Figure 1.**
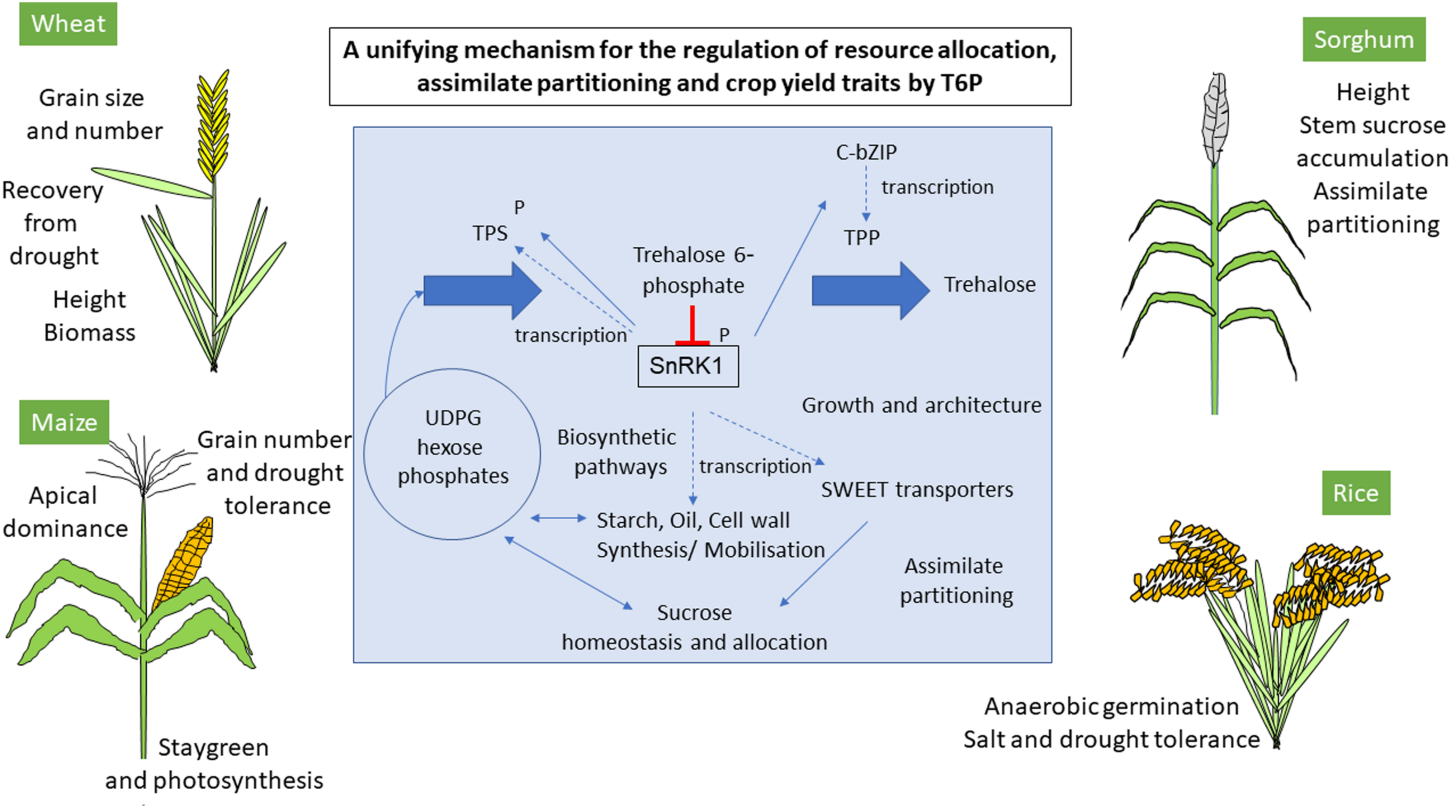
Mechanistic basis for the impact of T6P on growth, architecture and metabolism and demonstrated links to crops traits. T6P as a signal of sucrose inhibits SnRK1 to promote biosynthetic pathways [9] through change in SnRK1 phosphorylation [15]. SnRK1 regulates trehalose phosphate synthases (TPS) through transcription control [11] and phosphorylation [49] and transcription of TPPs through b-ZIP transcription factor [41]. Dotted lines in the figure denote transcription. There is evidence that SnRK1 regulates b-ZIPs [10]. SWEET transporters may be important downstream targets for the regulation of sucrose allocation [19]. Wide ranging traits in four food security cereals are associated with T6P as seen through transgenic modification in maize and rice [13,24], natural genetic variation in wheat [43,45], rice [26] and sorghum [40] and chemical intervention with T6P precursors in wheat [42].

It was subsequently shown that T6P, the direct precursor of trehalose, was indispensable for carbohydrate use in plants [8]; the indispensability shown to be through the feast/famine protein kinase, SnRK1 (sucrose non fermenting protein kinase [9,10]. SnRK1 is a member of the AMPK/ SNF1 group of protein kinases found in all organisms that regulate appropriate metabolic and developmental responses to carbon and energy availability [11]. T6P was shown to be a non-competitive inhibitor of SnRK1 with a Ki of 5 µM in Arabidopsis [9,12] and 50–60 µM in maize and wheat [13,14]. Later work has shown that T6P regulates the phosphorylation of SnRK1 by weakening its interaction with the SnRK1 upstream kinase GEMINIVIRUS REP-INTERACTING KINASE1 (GRIK1) decreasing phosphorylation resulting in less activation of SnRK1 [15]. G1P and G6P were also found to inhibit SnRK1 with Kis 480 µM and >1 mM, respectively [12,16] with indications of synergistic inhibition between T6P and G1P [12]. There is the potential therefore for T6P to inhibit SnRK1 more in biosynthetic tissues that accumulate substrates for the biosynthesis of end-products such as starch. T6P levels change much more (over a 1000-fold range) than G6P and G1P [17,18], hence T6P provides more dynamic regulation of SnRK1 than could be achieved by G1P or G6P alone. The main factor that regulates T6P levels is sucrose [17,18]; T6P is therefore regarded as a sucrose signal, however, the impact of sucrose on T6P is attenuated by development and cell and tissue type [14,28]. Through SnRK1, T6P de-represses gene expression for carbon use in biosynthetic pathways enabling growth and development to proceed under conditions of sufficient carbon availability [9,18]. Interestingly, T6P was shown necessary for the growth burst upon return to warm after a period of low temperature [18]. In the cold sugars accumulate increasing T6P impacting downstream T6P/SnRK1 marker gene expression; however cold still blocks the growth response that the altered gene expression might otherwise facilitate. Upon warming, however, after the cold, a growth spurt is observed, for which T6P was shown to be necessary through priming gene expression for growth in the cold due to sucrose and T6P accumulation [18]. This knowledge may enable the development of strategies to ensure better recovery of crops from abiotic stresses such as cold or drought. Contrasting approaches to the modification of T6P levels in crops could be taken. Elevating T6P would promote growth processes and biosynthesis of end products such as starch in grain and promote growth recovery after abiotic stress. In contrast, low T6P levels would stimulate catabolic and survival mechanisms through active SnRK1. The two opposing models of elevating T6P and lowering T6P could be targeted in different cells and tissues. On this basis, the pathway would seem an ideal candidate for potential modification to alter growth, development and architecture and the biosynthetic pathways that underpin accumulation of yield-determining end-products such as starch in productive and unproductive environments to combine yield with resilience (Figure 1).

## T6P signalling in crop improvement

**Figure 2.**
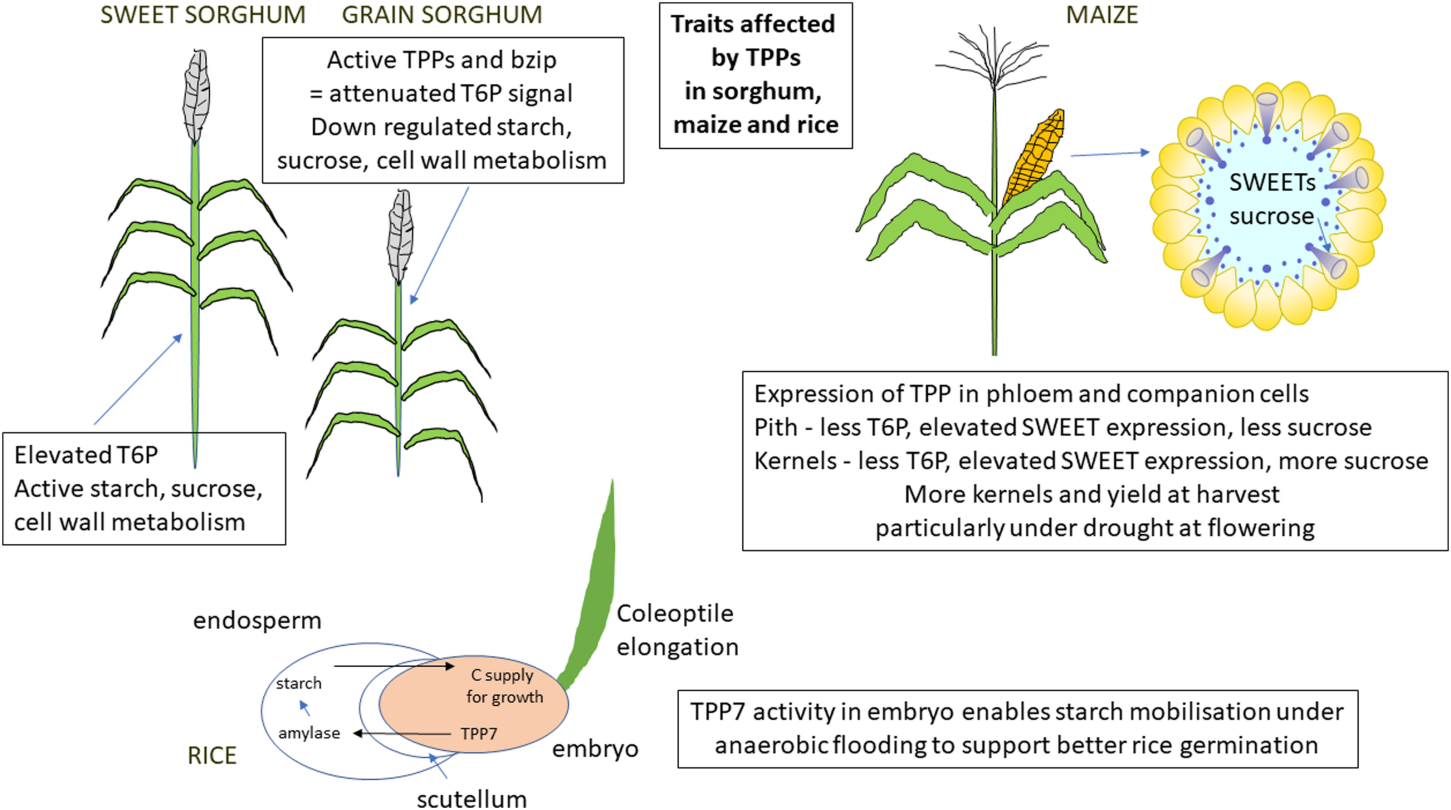
Examples of three important traits affected by T6P signalling in sorghum, maze and rice modulated by trehalose phosphate phosphatases. bZIP and TPP produce differential T6P signal and sucrose accumulation in sweet sorghum stems (high T6P) and down-regulated stem sucrose, starch and cell wall metabolism (low T6P in grain sorghum [40]. Overexpression of a rice TPP gene in maize alters sucrose flow within maize cob in favour of kernels [19]. A TPP gene in rice promotes starch mobilisation and better germination under flooding [26].

Four food security cereals, rice, maize, wheat and sorghum have all showed a decisive contribution of T6P signalling in crop traits (Figures 1 and 2). This review focuses on these crops because being top food security crops the global impacts in agriculture are likely to be largest and because examples in these crops are often underpinned by good mechanistic understanding of the basis of the T6P-dependent improvement. Modifying T6P signalling has been widely recognised as one important strategy in crop improvement [20]. More examples are likely to emerge from these food security cereals and other crops as knowledge of gene function, mode of action and the genetic and chemical tools to modify T6P develop. There are examples of association of individual enzymes of starch and sucrose metabolism with crop yield traits of cereal grain filling and size. For example, in maize and rice, cell wall invertase (*ZmINCW1* and *OsGRAIN INCOMPLETE FILLING1*) are associated with seed size and weight [21]. There is evidence that genes involved in starch metabolism *BT1*, [22] involved in ADP-glucose transport and sucrose synthase [23] have been selected in wheat improvement. The role of altered T6P signalling in these associations and selections is not known. However, given the role of T6P signalling in carbohydrate metabolism associated with crop traits as outlined below it is quite likely that changes in carbohydrate metabolism elicited by these enzymes may have been coordinated with changes in the T6P signalling pathway. Support for the view that significant changes in metabolism in crops will require changes in more than one enzyme plus the full integration of such changes with regulatory processes such as the T6P signalling pathway is illustrated by the lack of success in overexpressing individual enzymes of carbon metabolism in crops to improve yield in field conditions.

## Rice

Rice was the first major crop where transgenic modification of the trehalose pathway showed a real possibility in crop improvement. Garg et al. [24] expressed a fused *E. coli otsA* and *otsB* transgene in rice under the control of tissue-specific or stress-inducible promoters. The purpose of using a TPS-TPP gene fusion is to increase trehalose without necessarily increasing T6P. Plants showed better performance under drought, salt and cold stress. Low amounts of trehalose were present and plants accumulated more soluble carbohydrate and had elevated photosynthesis. However, the transgenic lines in this study do not appear to have progressed to commercial production. Improved tolerance to drought, salinity and sodic conditions (soil with an exchangeable sodium of >6% of the cation exchange capacity) was also shown using an ABA-inducible promoter to target the transgene [25]. In both studies, effects could have arisen through ROS scavenging and protection of cell membranes by trehalose. Alternatively, an effect on sugar signalling may have occurred. Kretzschmar et al. [26] showed a TPP gene, *OsTPP7*, as the genetic determinant in qAG-9-2, a major quantitative trait locus (QTL) for anaerobic germination tolerance. In this case, it is thought that lowering of T6P levels in germinating embryo and elongating coleoptile enhances mobilisation of starch fuelling better germination under anaerobic conditions (Figure 2). The rice SUB1A-1 ethylene transcription factor gene confers tolerance to prolonged complete submergence, by limiting rather than promoting underwater elongation growth in contrast with qAG-9-2. Expression of TPS and TPP genes is altered in SUB1A compared with control suggesting involvement of the pathway in the management of energy metabolism [27]. However, T6P levels did not differ in SUB1A compared with control so exact involvement of T6P in limiting underwater growth in SUB1A is not clear. *OsTPS8* (*OS08G0445700*) was shown to confer salt stress tolerance by enhancing suberin deposition in roots [28]. Suberin is part of a hydrophobic barrier that blocks apoplastic leakage of ions into the xylem and damaging accumulation of Na^+^ in shoots. It was proposed that *OsTPS8* regulates suberin deposition through ABA signalling. Although given the known effect of T6P/SnRK1 on lipid metabolism [15,19] and that suberins contain fatty acids, T6P/SnRK1 could also be involved potentially in the regulation of suberin synthesis. *GROWTH REGULATING FACTOR (GRF4*) is a transcription factor that promotes and integrates nitrogen assimilation, carbon fixation and growth, counterbalancing the inhibiting action of DELLAs of the gibberellin signalling pathway. It was shown that *GRF4* up-regulates TPS and TPP genes and SWEET sucrose efflux transporters as part of the growth-promoting process [29]. Such interactions with T6P signalling could be a common feature given the need to coordinate sucrose supply and metabolism with the regulation of growth and development.

## Maize

*RAMOSA3* controls inflorescence architecture in maize and was found to be encoded by a TPP gene which regulates meristem fate through catalytically inactive TPPs also including *TPP4* [30,31]. No effect of these TPPs on T6P has been proven and a moonlighting role for *TPP4* has been put forward; the exact mechanism through which meristem fate is regulated by TPPs is not known. Whole-plant architecture in terms of apical dominance appears to have been strongly influenced in maize by the *TEOSINTE BRANCHED* (*TB1*) transcription factor. *TB1* targets are *TPS2*, *TPP1*, *RAMOSA3* and *TREHALASE1* which were found to be altered in buds of a *tb1* mutant. This could mean that in maize, as in pea [32], T6P is involved in the regulation of bud outgrowth and apical dominance and that this has been selected during the domestication process through *TB1* in maize [33]. In other studies in maize, the expression of TPS and TPP genes responds strongly to darkness [34,35] and salt stress [36] showing a likely central involvement in energy metabolism and carbon management under these stresses.

Nuccio et al. [13] and Oszvald et al. [19] presented a very detailed field and phenotypic analysis of transgenic maize overexpressing a rice TPP gene. It was an exemplar study for the extent of analysis required to prove efficacy of a new transgenic variety in a grower environment. Extensive field trialling in different locations showed that overexpression of the *OsTPP1* gene in maize improved yield under a range of water availabilities. In this instance over expression of the TPP gene with a MADS6 promoter active during the flowering period in the phloem of female reproductive tissue, particularly companion cells, reduced T6P levels in pith tissue (Figure 2; [19]). This was associated with up-regulation of sucrose efflux regulators, SWEETs, which could explain enhanced sucrose flow from pith to developing kernels [19]. Interestingly, SWEETs are also associated with a change in T6P in the control of bud outgrowth in *tb1* maize (above). The important points [13] are firstly the careful targeting of expression of TPP and hence T6P contents in phloem vasculature and companion cells during the flowering period [19]. Many other constructs were tried with slightly different expression profiles which were not effective in improving yield. Secondly, improving sucrose flow to kernels is a means to prevent kernel abortion and improve yield under drought and SWEETs were key in this. Thirdly, changing T6P in reproductive tissue resulted in maintenance of a higher rate of photosynthesis for longer in leaves. This shows the whole-plant reach of T6P with strong source-sink regulation in the form of sucrose movement from pith to kernels in reproductive tissue affecting photosynthesis distally in leaves. Interestingly, leaf senescence was found related to T6P levels and SnRK1 transcript [37] and a stay-green trait has been associated with maize *TPS13* (Zm00001d020396) [38] in confirmation of earlier work [39] that showed a role of T6P in leaf senescence in Arabidopsis. Fourthly, Oszvald et al. [19] showed differential assimilate partitioning accumulation within a sink organ: more sugars, amino acids and lipids in kernels, less in pith tissue. Interestingly, the study showed different effects of T6P on primary metabolism (low T6P down-regulated primary metabolism as expected) and secondary metabolism (low T6P up-regulated secondary metabolism). This shows the potential to alter partitioning between primary and secondary metabolism. Fifthly, the work confirmed the role for T6P/ SnRK1 as a central mechanism of resource allocation with effects on SnRK1 activity, SnRK1 marker genes as well as endogenous TPP and TPS gene expression. In a recent study in sorghum (below), assimilate partitioning was also shown to be decisively affected by T6P.

## Sorghum

Sorghum, grown in tropical and sub-tropical regions, is the fifth most important cereal worldwide after maize, rice, wheat and barley. Li et al. [40] published an important study that showed that T6P/SnRK1 could play a key role in the diverse assimilate partitioning patterns of sweet and grain sorghum (Figure 2). Sweet sorghum is taller than grain sorghum and accumulates large amounts of sugar in the stem which gives it uses as a bioenergy crop. Sweet sorghum was found to have active sucrose, starch and cell wall metabolism in stems with accumulation of sucrose and sugar phosphates compared with grain sorghum where these pathways were down-regulated and stem accumulation of sugars and metabolites was far lower. In a cross between sweet and grain sorghum it was found that TPP genes and bZIP transcription factor that controls expression of TPPs [41] derived from the grain sorghum parent were linked to lower T6P levels in the progeny which could account for the down-regulation of sucrose, starch and cell wall metabolism in stems. Elevated TPP activities, the expression of which can be controlled by bZIP transcription factor could decrease T6P. T6P/SnRK1 inducible or repressible genes were significantly enriched up or down in transcriptomics in alignment with T6P levels. Induction of sucrose, starch and cell wall metabolic pathways in sweet sorghum was associated with higher T6P and induction of transcripts for these pathways. Understanding the mechanistic basis of assimilate partitioning in plants has been a grand challenge. This is the first extensive study building on [19] that links T6P/SnRK1, or indeed any plausible mechanism to assimilate partitioning and the differential accumulation of end products in crops and is an important advance. Such findings may be applicable to crops generally beyond stem sugar accumulating crops.

## Wheat

Martinez Barajas et al. [14] presented the first characterisation of T6P levels in a major food security crop. The striking observations from these results were firstly the high levels of T6P observed up to 119 nmol g^−1^ fresh weight relative to Arabidopsis seedlings (0.01 nmol g^−1^ fresh weight in sugar-starved seedlings [17] up to 10 nmol g^−1^ fresh weight in cold treated seedlings [18]) and secondly the large differences within grain tissues over the course of development. There was a transition from high T6P levels in both maternal (pericarp) (47–117 nmol g^−^1 fresh weight) and paternal tissue (endosperm) (119 nmol g^−1^ fresh weight) at 7 days after anthesis (DAA), to high levels in endosperm only at 17 DAA during grain filling. High T6P levels in endosperm may be associated with driving starch synthesis during grain filling. In confirmation of this, Griffiths et al. [42] sprayed wheat ears at 10 DAA with light-labile T6P precursors that can enter cells to release a large pulse of T6P upon exposure to bright light. This increased grain size, starch content and yield by up to 20% and was associated with an increase in expression of genes of starch synthesis in the grain. In confirmation of an effect of T6P on grain size, genetic variation in a TPP gene has been associated with thousand grain weight and explaining 12.1–19.1% of the phenotypic variance across five environments [43]. Interestingly, [42] also showed the potential of light-labile T6P precursors in growth recovery after drought stress in support of the view that T6P can prime gene expression for growth prior to the stressor — drought [42] or cold [18] being removed.

**Figure 3.**
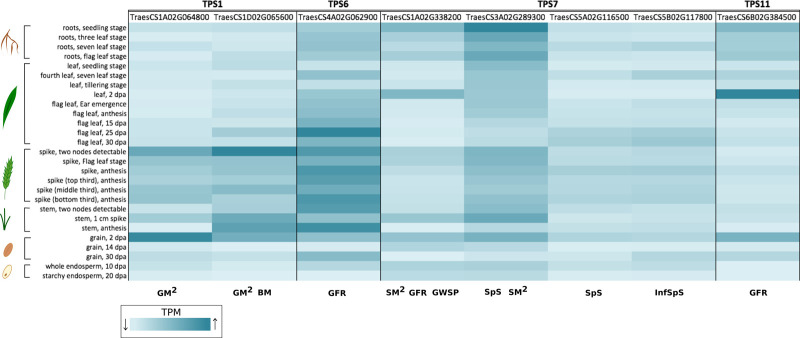
Developmental expression of key wheat TPS genes associated with yield components in the HiBAP panel from Table 1 [45]. Transcripts per million (TPM) reads for each gene of interest were extracted from the wheat exvip server from the listed plant organs [50,51]. All genes chosen where associated with SNPs related to GM2 (grain number per m^2^), BM (biomass), GFR (grain filling rate), SM2 (spikes per m^2^), GWSP (grain weight per spike), SpS (spikelets per spike), InfSpS (infertile spikelets per spike) as listed at the bottom of the heatmap (dark blue (high transcript per million, TPM) — light blue (low TPM)).

**Figure 4.**
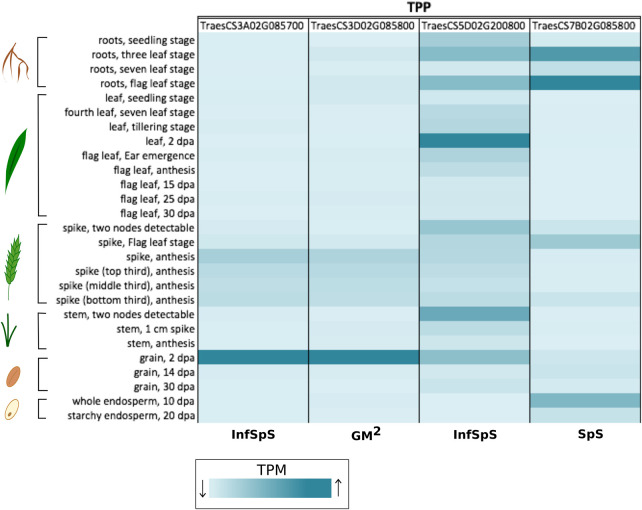
Developmental expression of key wheat TPP genes associated with yield components in the HiBAP panel from Table 1 [45]. Transcripts per million (TPM) reads for each gene of interest were extracted from the wheat exvip server from the listed plant organs [50,51]. All genes chosen where associated with SNPs related to GM2 (grain number per m2), SpS (spikelets per spike), InfSpS (infertile spikelets per spike) as listed at the bottom of the heatmap (dark blue (high TPM) — light blue (low TPM)).

Trehalose pathway genes were listed as domestication improvement candidates in maize [44], however, the link to traits was not shown. It leads to the interesting question of the extent to which domestication and breeding have already directed selection of TPS and TPP variants, for which traits and to what extent further changes are possible. The interventions so far discussed here in different crops indicate that further changes in the trehalose pathway are indeed possible for crop improvement beyond what breeding and selection have already achieved. Up until now a comprehensive evaluation of genetic variation in TPS and TPP genes and link to traits had yet to be performed for a major crop. Lyra et al. [45] used exome-capture sequencing on TPS and TPP genes from [46] to estimate and partition the genetic variation of yield-related traits in a spring wheat high biomass associated breeding panel (HiBAP [47]). Two analyses were carried out with the sequences of TPS and TPP genes to look for associations with several traits measured on the same population at the gene-level and at the level of SNPs within these genes. Table 1 summarises the significant gene-trait associations that were found. Expression of these genes was wide ranging in roots, leaves and reproductive tissue (Figures 3 and 4). In field trials under fully irrigated conditions in Yaqui Valley, Mexico, twelve phenotypes were directly correlated to TPS and TPP genes including biomass, plant height, spikelet fertility, spikes per m^2^, grains per m^2^ and grain-filling traits. Interestingly, some genes for traits were under positive selection indicating that genetic change in both TPS and TPP genes was being tolerated and selected into new material e.g. for grains per m^2^, plant height, infertile spikelets per spike and grain filling duration. Some genes also showed a high degree of heritability for grain per m^2^, grain per spike, grain filling duration, biomass and days to anthesis indicating a strong contribution of the pathway to traits. Not surprisingly in a pathway where TPS and TPP act together there were epistatic interactions between TPS and TPP genes e.g. between TPS1 and a TPP gene and also within TPS and TPP genes. The data indicated a considerable contribution of this regulatory pathway to phenotypic variation, showing historical selection for harvest index, final biomass, plant height and flowering time with further opportunities to improve traits that increase grain numbers per unit area of land and grain filling which would likely affect grain size. The paper provides a basis for greater in-depth characterisation of these genes in yield traits and for use in strategic crosses and is likely to be an important resource of information for the improvement of wheat and possibly other crops too.

**Table 1:** Comparison or Arabidopsis trehalose phosphate synthases (TPS) and trehalose phosphate phosphatases (TPP) with rice, maize and wheat TPSs and TPPs

Arabidopsis (2n = 2x = 10)	Rice (2n = 2x = 24)	Maize (2n = 2x = 20)	Wheat (2n = 6x = 42)
**Trehalose phosphate synthase**			
Class I TPS TPS1, 2, 3, 4	1 gene	2 genes	7 genes TraesCS1A02G064800 GM2 TraesCS10D01G065600 GM2, PH, biomass
Class II TPS5	0	0	0
TPS6	2 genes	2 genes Zm00001d020396 Association with stay green [38]	6 genes TraesCS4A02G062900 GFR
TPS7	3 genes	4 genes improvement candidates [44]	9 genes TraesCS1A02G338200 SM2, GFR, GWSP TraesCS3A02G289300 SpS, SM2 TraesCS5A02G116500 SpS TraesCS5B02G117800 InfSpS
TPS8, 9, 10	2 genes	3 genes improvement candidates [44]	1 gene
TPS11	3 genes OsTPS8, salt stress tolerance [28]	5 genes	2 genes TraesCS6B02G384500 GFR
Total 11	11 genes	16 genes	25 genes
**Trehalose phosphate phosphatase**			
1 TPPA, F, G	2 genes	2 genes	3 genes
2	2 genes	4 genes ramosa-3 meristem determinancy [30]	9 genes
3	2 genes OsTPP7, flooding tolerance [26]	3 genes improvement candidates [44]	3 genes TraesCS5D02G200800 InfSpS
4	1 gene	3 genes ZmTPP4 meristem determinancy [31]	6 genes TraesCS7B02G085800 SpS
5 TPPB, C, D, E, H, I, J	0	0	0
6	0	0	4 genes TraesCS3A02G085700 InfSpS
7	2 genes	1 gene OsTPPP1 overexpression improves drought tolerance [13]	6 genes TraesCS6A01G248400 Grain weight [43]
Total 10	10 genes	13 genes	31 genes

Numbers of the respective genes are depicted for each species. Genes are from phylogenetic analysis of TPSs and [46]. TPP genes rice, maize and wheat genes diverge from Arabidopsis TPPs and are numbered as clades clockwise from clade 1 (TPPA, F, G) from [46]. Specific genes are linked to traits where there is published evidence. *OsTPS8* and *OsTPP7* are [28] and [26], respectively. Improvement candidates in maize are from [44]. *RAMOSA3* and *ZmTPP4* are published in [30,31], respectively. *OsTPP1* overexpression in maize improves grain numbers and yield under drought at flowering [13]. Wheat TPSs and TPPs have been linked to several traits [45]. Those listed here are significantly linked to yield related traits in [45]. GM2 is grains per m^2^; PH is plant height; GFR is grain filling rate; SM2 is spikes per m^2^; GWSP is grain weight per spike; SpS is spikelets per spike; InfSpS is infertile spikelets per spike. TraesCS6A01G248400 has been linked to grain weight [43].

## Conclusion and outlook

It has taken 20 years of research to go from interesting observations in Arabidopsis and tobacco to a translation of the knowledge of T6P signalling into crop improvement. We are now at the point where research in crops can go to the next level through targeting T6P in different ways (genetics and chemistry) to achieve tangible benefits for both yield potential and yield resilience in a range of crops. It will be important to understand the mode of action of T6P action through the T6P/SnRK1 system i.e. the cells in which the mechanism achieves the effects on yield traits and the downstream gene targets and how this has been changed over the course of breeding and selection. TPP genes particularly in cereals appear to be very different from Arabidopsis [46]. Interestingly, so far TPP genes have been associated with the most significant crop traits in cereals (Figure 2). Knowledge of the molecular mechanism of how T6P levels are regulated is still unclear together with the function of the TPS and TPP genes and the SnRK1 and protein complexes involved in the T6P/SnRK1 mechanism. Such knowledge could help refine crop improvement by T6P and provide strategies to modify T6P levels up or down and or alter the perception and signalling of T6P. There are of course other genes in source and sinks that have shown utility in crop improvement particularly in hormone signalling pathways and several transcription factors already mentioned [20,29,48]. It will be interesting to better understand how the T6P pathway interacts with, is regulated by, or regulates any of these other systems. It is likely to take another 20 years for the full benefits of modifying T6P signalling in crops to be realised, but this should be a very exciting period. T6P signalling has undoubtedly already been selected for in crops, but further significant benefits have been shown possible in food security cereals. In less advanced and orphan crops where a limited selection of the pathway may have occurred large advances could be made in targeting the pathway.

## Perspectives

- **Importance of T6P signalling in crops:** For the improvement of crop yields there are likely to be few individual genes that can be targeted, yet for T6P signalling there are examples of several TPS and TPP genes that underlie yield traits [13,25,43,45]. Further confirmation of T6P as a major regulator of yield was shown where increasing T6P chemically in wheat improved grain size and growth recovery from drought [42]. The reason for the large effect of the T6P signalling pathway on traits in crops is because T6P regulates carbon allocation for metabolic pathways, assimilate partitioning, growth, development and architecture which underpins all crop processes.
- **A summary of current thinking:** The inhibition of SnRK1 by T6P [9] where sucrose via T6P provides carbon resource input in a central carbon and energy sensor provides the basis for a unifying mechanism controlling whole-plant resource allocation and source-sink interactions in crops. Up until now a generic mechanism for whole-plant carbon resource allocation has been unknown. Further details await elucidation e.g. exactly which genes downstream of T6P/SnRK1 are important for particular crop traits. SWEET genes may be particularly important in the T6P regulation of sucrose transport and allocation.
- **Future directions:** For crop improvement, there are numerous avenues for modifying and selecting T6P signalling in crops. Transgenic approaches have shown good effects in rice and maize. However, the difficulties and regulatory costs associated with getting new transgenic varieties to the marketplace and lack of wide acceptance may prove the transgenic approach to be difficult. Gene editing does offer new promise combined with selection of TPS and TPP genes positively linked to traits [45]. Additionally, chemical methods that can perturb T6P *in vivo* at any developmental time point provide a new way to increasing both yield potential and resilience through growth recovery after stress [42]. Mode of action studies of downstream of T6P/SnRK1 are important because this can identify other interacting factors and genes that can be combined with modification of T6P or in themselves become targets.

## Abbreviations

SnRK1: sucrose nonfermenting related protein kinase1
T6P: trehalose 6-phosphate
TPP: trehalose phosphate phosphatase
TPS: trehalose phosphate synthase
